# Myocardial infarction with non-obstructive coronary arteries in a patient double-seropositive for anti-glomerular basement membrane and anti-neutrophil cytoplasmic antibodies: A case report

**DOI:** 10.3389/fcvm.2022.893742

**Published:** 2022-09-23

**Authors:** Marcell Krall, Johannes Gollmer, Marion J. Pollheimer, Clemens Reiter, Michael Kolland, Alexander H. Kirsch, Andreas Kronbichler, Kathrin Eller, Alexander R. Rosenkranz, Balazs Odler

**Affiliations:** ^1^Division of Nephrology, Department of Internal Medicine, Medical University of Graz, Graz, Austria; ^2^Division of Cardiology, Department of Internal Medicine, Medical University of Graz, Graz, Austria; ^3^Institute of Pathology, Medical University of Graz, Graz, Austria; ^4^Division of General Radiology, Department of Radiology, Medical University of Graz, Graz, Austria; ^5^Department of Medicine, University of Cambridge, Cambridge, United Kingdom

**Keywords:** MINOCA, ANCA, anti-GBM, double-positive, coronary, therapy

## Abstract

We report a case of a patient double-seropositive for anti-glomerular basement membrane (anti-GBM) and anti-neutrophil cytoplasmic antibodies (ANCA) who reported retrosternal chest pain during a regular hemodialysis session associated with ST-segment depression in electrocardiogram and an increase of serum high-sensitivity troponin T. Urgent coronary angiography excluded obstructive coronary artery disease, suggesting the diagnosis of ischemia with non-obstructive coronary arteries. This case illustrates an unusual presentation of cardiovascular involvement in a patient with double-positive ANCA/anti-GBM disease, emphasizing the possible relevance of coronary microvascular dysfunction and the need for close cardiovascular follow-up in this patient population.

## Introduction

Anti-neutrophil cytoplasm antibody (ANCA)-associated vasculitides (AAV) and anti-glomerular basement membrane (GBM) disease are rare entities (1, 2). The coincidence of positivity for anti-GBM and ANCA antibodies with clinical features of both diseases represents a life-threatening entity (3). Cardiovascular (CV) events are among the leading causes of death in AAV; thus, a more complex understanding of CV manifestations and their risk factors is required (4). In line, cardiac involvement is an underdiagnosed manifestation in AAV, and involvement of the coronary arteries is only rarely reported (5–9). Our case illustrates that myocardial infarction with non-obstructive coronary arteries (MINOCA) might be an important contributor to cardiac complications seen in patients with AAV and/or anti-GBM disease.

## Case presentation

**FIGURE 1:**
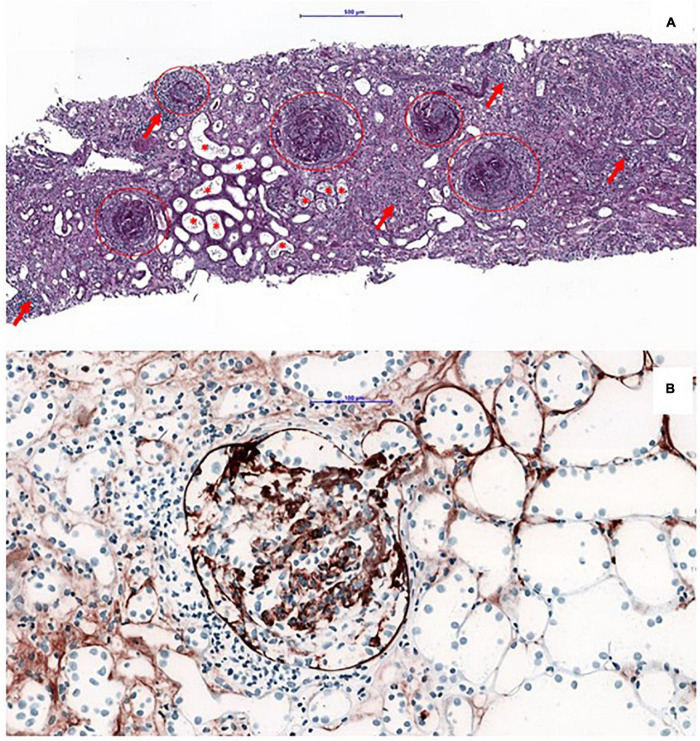
Kidney biopsy: an overview of a PAS-stained kidney biopsy, showing large cellular crescent present in all glomeruli (as highlighted by the circles), in line with a severe presentation of anti-glomerular basement membrane (anti-GBM) disease. Concomitant tubulo-interstitial nephritis (arrows) and signs of severe acute tubular injury with red blood cells within the tubular lumina (asteriks). Bar = 500 μm **(A)**. Immunohistochemical staining with the antibody against IgG, showing a linear staining pattern of the glomerular basement membrane **(B)**.

A 61-year-old Caucasian man presented to our university clinic with acute kidney injury. He was diagnosed with scleritis of the right eye approximately 13 years before current admission and had received azathioprine (AZA) for immunosuppression due to suspected myeloperoxidase (MPO)-ANCA positive granulomatosis with polyangiitis (GPA) without evidence of further organ involvements. Besides, he had arterial hypertension treated with angiotensin-converting enzyme inhibitor/hydrochlorothiazide and had been diagnosed with prostate cancer 2 years before current admission (treated by radiotherapy and gonadotropin-releasing hormone analog). The AZA therapy had been discontinued 19 months prior to presentation due to an influenza infection. Thereafter, on admission, a nephritic syndrome was present. Laboratory investigations revealed an increase of MPO-ANCA and anti-glomerular basement membrane (anti-GBM) antibody titers (>100 and 244 U/ml, respectively). Thus, a kidney biopsy was performed, and the diagnosis of double-positive disease was made (Figures 1A,B).

Plasma exchange (14 sessions), high-dose corticosteroids, and cyclophosphamide (CYC – cumulative dose of 3.3 g) were initiated. However, he progressed to end-stage kidney disease and required hemodialysis (HD). In addition, 2 doses of rituximab (RTX) 500 mg each 2 weeks apart were given followed by 500 mg every 6 months as maintenance immunosuppressive therapy.

**FIGURE 2:**
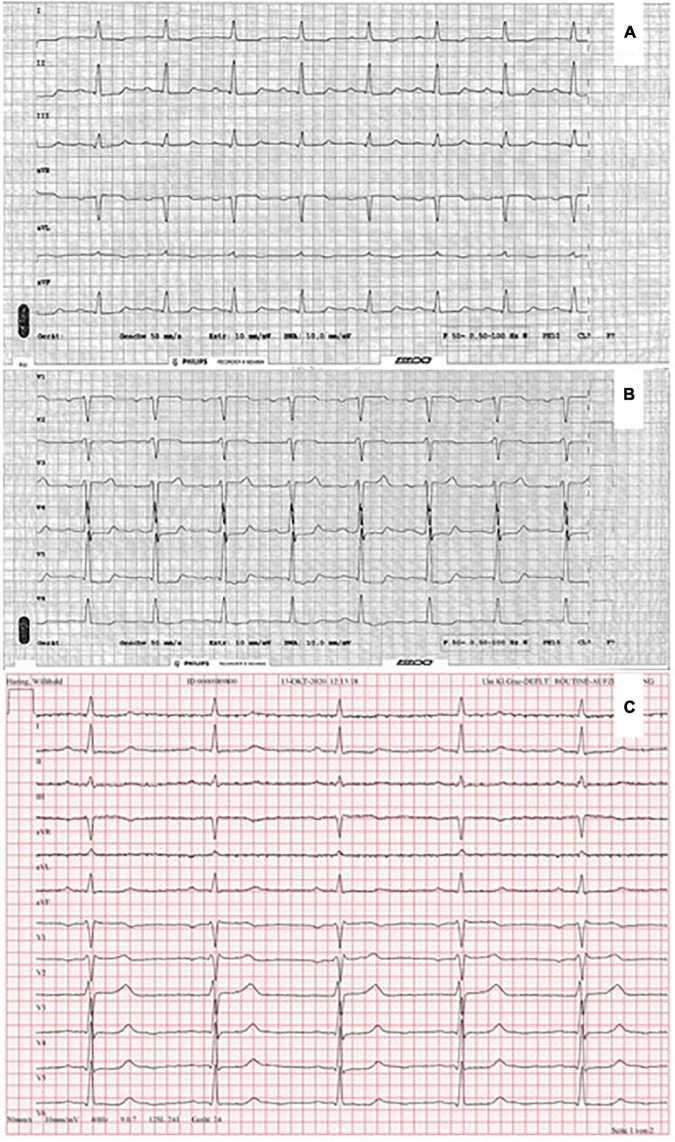
Electrocardiogram showing ST-segment depression in II, III, aVF **(A)**, and V4–V6 **(B)**. Electrocardiogram (with a paper speed of 50 mm/s) 1 month before MINOCA **(C)**.

**FIGURE 3:**
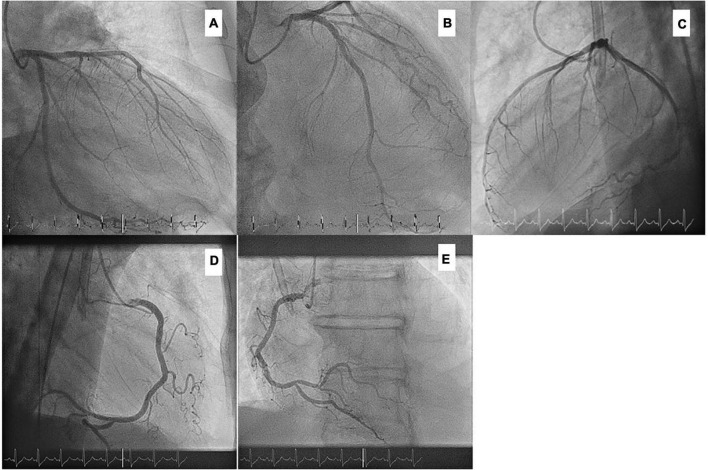
Coronary angiography without significant obstructive coronary artery disease. **(A)** cranial, 40° **(B)** RAO, 20°; caudal, 30° **(C)** LAO, 90° **(D)** RAO, 90° **(E)** LAO, 30°.

**FIGURE 4:**
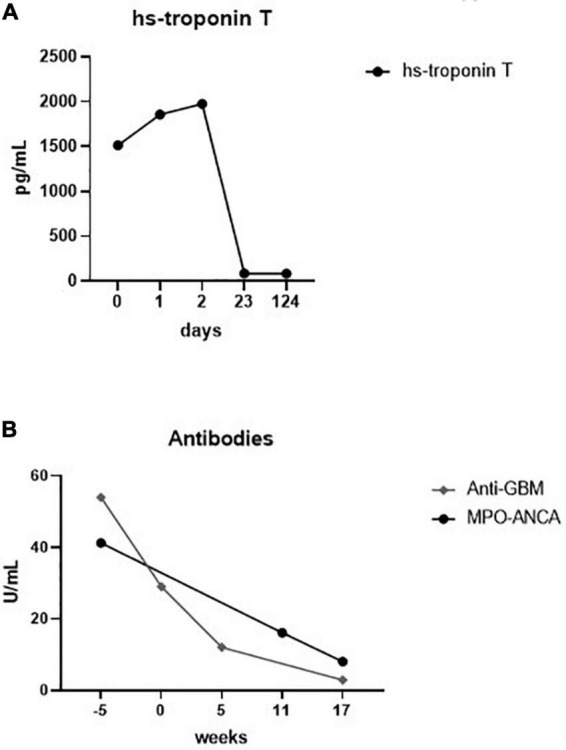
Laboratory charts of the time-dependent development of high-sensitive (hs) troponin T **(A)** and myeloperoxidase anti-neutrophil cytoplasm (MPO-ANCA) and anti-glomerular basement membrane (anti-GBM) antibodies **(B)**. Zero on both scales indicates the time point of chest pain.

After 4 weeks of HD initiation, during a regular HD session, the patient reported retrosternal chest pain. Electrocardiogram (ECG) showed ST depressions in leads II, III, aVF, and V4 to V6 (Figures 2A,B), which changes were absent in a previous ECG 1 month before (Figure 2C). High-sensitive troponin T (hs-Tn) was elevated (the baseline level: 1,514 pg/ml; normal range: <14 pg/ml); thus, the diagnosis of non-ST elevation myocardial ischemia was made (Figure 3). Bed-side echocardiography revealed mildly reduced ejection fraction (40%) with diffuse hypokinesia and pronounced abnormalities in inferior, inferolateral, and anterolateral segments. Urgent coronary angiography (CA) excluded obstructive coronary artery disease (CAD) in major epicardial vessels and did not show signs of ruptured plaque or dissection, suggesting the diagnosis of MINOCA (Figures 3A–E). Cardiac magnetic resonance (CMR) imaging was performed to exclude acute (peri) myocarditis. Cine imaging revealed severe left ventricular and atrial dilatation with global hypokinesia and reduced left ventricular ejection fraction, aortic and mitral insufficiency but preserved right ventricular function. Native myocardial T1-values were globally elevated, myocardial T2-values were within the normal ranges, T2-weighted images showed no signs of edema, and no late enhancement was visualized, therefore not fulfilling diagnostic criteria of an acute myocarditis (10) but consistent with diffuse myocardial fibrosis in dilated cardiomyopathy. Myocarditis was also ruled out by positron emission computed tomography. Notably, ANCA positivity was seen throughout the disease course, while hs-Tn decreased significantly a few weeks after the cardiac event (Figure 4).

## Discussion

Double-positive disease is a life-threatening disorder characterized by small vessel vasculitis affecting the kidneys and the lungs with a coincidence of anti-GBM disease and AAV (11). Cardiac involvement is a known disease manifestation in AAV, predominantly in those with MPO-ANCA positivity, and associated with increased mortality (12). On the contrary, CV complications are rarely reported in patients with anti-GBM disease. Only one case of anti-GBM disease with cardiac involvement was reported in our literature review (13).

Myocardial infarction with non-obstructive coronary arteries represents a major health problem and is often misdiagnosed, leading to possible undertreatment (14). Patients with small-vessel vasculitis have a high CV event rate, even exceeding high-risk populations, such as those with chronic kidney disease (4). Recently, there has been an increasing interest to identify patients at risk since they have a higher risk of developing major cardiovascular events and being hospitalized due to heart failure or repeated CA (15–17). Adequate diagnosis is challenging; however, if clinical surrogate markers of myocardial ischemia without evidence of obstructive CAD are present, the diagnosis of MINOCA can be considered (18). Dilated cardiomyopathy as a possible cause of chest pain in our patient cannot be excluded entirely. However, he had typical angina pectoris with dynamic ischemic ECG changes and a high-serum level of hs-Tn (again, later falling despite chronic HD), while invasive angiography did not reveal coronary stenosis (≥50%). In accordance, the absence of myocarditis and tear on angiography also underlines the clinical diagnosis of MINOCA. Importantly, the use of CYC is a considerable risk factor in cardiotoxicity; however, its triggering minimum dose is not known. Registry data reveal an association between high-dose CYC (>36 g) and ischemic heart disease in patients with GPA (19).

A study by Pugnet et al. analyzed patients with GPA who underwent CMR (20). In patients, who had simultaneous CA with normal coronary findings, subendocardial late gadolinium enhancement (LGE) with perfusion defects, suggesting ischemic lesions possibly due to small vessel vasculitis, was seen. In our case, CMR showed no late enhancement, suggesting the absence of inflammation.

Mechanisms contributing to the development of MINOCA seem to be heterogeneous but characterized mainly by coronary vasospasm and microvascular dysfunction (14, 21). In our patient, invasive functional CA and pharmacological reactivity testing was not performed; thus, the contributing mechanism behind his symptoms remains unclear. Nevertheless, strong evidence on the association between coronary vascular dysfunction and small vessel disease exists (22). In patients with AAV, ANCA-activated neutrophils induce endothelial injury, which might lead to coronary vascular dysfunction and promote the induction of epicardial vessel spasm in predisposed coronary segments. In addition, the endothelial impairment resulting in altered vascular tone enhances smooth muscle contractility and might be responsible for myocardial ischemia *via* decreased coronary flow and perfusion pressure. Moreover, similar to patients with systemic lupus erythematosus, systemic inflammation in AAV patients might also contribute to coronary vasomotor abnormalities (23). A recent European multicenter study has found that patients with double-positive disease have a relapse rate comparable to patients with AAV alone, suggesting the importance of long-term follow-up and maintenance immunosuppressive therapy in this patient population (11). In our patient, a preexisting extrarenal vasculitis with continuous ANCA positivity might be a possible endogenous contributor responsible for his myocardial ischemia besides the predominant clinical phenotype of anti-GBM disease.

As coronary microvascular dysfunction might have a significant impact on the patients’ quality of life as well as a clinical outcome, sufficient therapeutic strategies are of particular interest (24, 25). Since – to our knowledge – no studies are published dealing with this entity in patients with AAV and/or double-positive ANCA/anti-GBM disease, it is unclear which medical treatment might be the most sufficient. Recent major guidelines issued by the leading cardiology societies have provided a guidance on various pharmacological treatment options of MINOCA related to the underlying mechanism (14, 26). Accordingly, angiotensin-converting enzyme inhibitors or angiotensin receptor blockers might depict a useful option to reduce systemic inflammation and endothelial dysfunction (27). Calcium antagonists might be used in patients with evidence of either epicardial or microvascular spasm who underwent acetylcholine testing (14). As patients with AAV face increased CV risk even in the early phase of the disease, adequate lifestyle changes and rigorous CV risk management are of particular importance (28, 29).

There are some clear limitations to this presented case that merit notification. Invasive testing of coronary artery spasm and microvascular dysfunction, which might have provided direct evidence on some of the possible mechanisms of MINOCA, have not been performed in this case; therefore, MINOCA remains a “working diagnosis.” Also, certain underlying conditions, such as coronary thrombosis with a quick spontaneous lysis of the thrombus or uncontrolled blood pressure, cannot be fully excluded. Finally, our patient was on dialysis, and, thus, the contribution of prominent accumulation of uremic toxins over time as a specific trigger of microvascular dysfunction might also be occurred.

## Conclusion

In summary, we report a case of a patient double-seropositive for anti-GBM and ANCA antibodies with extrarenal vasculitis who presented with MINOCA. Now, studies improving our understanding of underlying mechanisms associated with excessive CV mortality in AAV patients are essential. Coronary microvascular dysfunction or vasospastic angina due to endothelial injury and/or systemic inflammation might be a significant contributor of MINOCA in patients with AAV and/or double-positive disease, who can be at higher risk to develop this condition. Given the increased risk of adverse CV outcomes associated with AAV, this entity might be an important and reversible cause, which needs a multidisciplinary care approach and further exploration in addition to traditional CV risk factors.

## Data availability statement

The original contributions presented in this study are included in the article/supplementary material, further inquiries can be directed to the corresponding author.

## Ethics statement

The studies involving human participants were reviewed and approved by the Ethics Committee of the Medical University of Graz. The patients/participants provided their written informed consent to participate in this case study. Written informed consent was obtained from the individual(s) for the publication of any potentially identifiable images or data included in this article.

## Author contributions

MaK and BO wrote the first draft of the manuscript. JG described the coronary angiography and ECG. CR evaluated and interpreted the cardiac MRI findings. MP performed the histological work-up. MaK, AKi, AKr, KE, and AR contributed to the data acquisition and drafting of the manuscript. All authors contributed to the article and approved the submitted version.
